# Determining the mode of action of anti-mycobacterial C17 diyne natural products using expression profiling: evidence for fatty acid biosynthesis inhibition

**DOI:** 10.1186/s12864-016-2949-y

**Published:** 2016-08-11

**Authors:** Haoxin Li, Andrew Cowie, John A. Johnson, Duncan Webster, Christopher J. Martyniuk, Christopher A. Gray

**Affiliations:** 1 Department of Biological Sciences, University of New Brunswick, PO Box 5050, 100 Tucker Park Road, E2L 4L5 Saint John, NB Canada; 2 Department of Medicine, Division of Infectious Diseases, Saint John Regional Hospital, 400 University Ave, E2L 4L4 Saint John, NB Canada; 3 Present address: Center for Environmental and Human Toxicology & Department of Physiological Sciences, UF Genetics Institute, College of Veterinary Medicine, University of Florida, 1333 Center Drive, 32610-0144 Gainesville, FL USA; 4 Department of Chemistry, University of New Brunswick, PO Box 4400, 30 Dineen Drive, E3B 5A3 Fredericton, NB Canada

**Keywords:** Gene network analysis, Natural products, Mode of action, *Mycobacterium smegmatis*, Falcarinol, Panaxydol

## Abstract

**Background:**

The treatment of microbial infections is becoming increasingly challenging because of limited therapeutic options and the growing number of pathogenic strains that are resistant to current antibiotics. There is an urgent need to identify molecules with novel modes of action to facilitate the development of new and more effective therapeutic agents. The anti-mycobacterial activity of the C17 diyne natural products falcarinol and panaxydol has been described previously; however, their mode of action remains largely undetermined in microbes. Gene expression profiling was therefore used to determine the transcriptomic response of *Mycobacterium smegmatis* upon treatment with falcarinol and panaxydol to better characterize the mode of action of these C17 diynes.

**Results:**

Our analyses identified 704 and 907 transcripts that were differentially expressed in *M. smegmatis* after treatment with falcarinol and panaxydol respectively. Principal component analysis suggested that the C17 diynes exhibit a mode of action that is distinct to commonly used antimycobacterial drugs. Functional enrichment analysis and pathway enrichment analysis revealed that cell processes such as ectoine biosynthesis and cyclopropane-fatty-acyl-phospholipid synthesis were responsive to falcarinol and panaxydol treatment at the transcriptome level in *M. smegmatis*. The modes of action of the two C17 diynes were also predicted through Prediction of Activity Spectra of Substances (PASS). Based upon convergence of these three independent analyses, we hypothesize that the C17 diynes inhibit fatty acid biosynthesis, specifically phospholipid synthesis, in mycobacteria.

**Conclusion:**

Based on transcriptomic responses, it is suggested that the C17 diynes act differently than other anti-mycobacterial compounds in *M. smegmatis*, and do so by inhibiting phospholipid biosynthesis.

**Electronic supplementary material:**

The online version of this article (doi:10.1186/s12864-016-2949-y) contains supplementary material, which is available to authorized users.

## Background

Despite significant progress by the World Health Organization, tuberculosis (TB) remains a global health emergency with millions of patients succumbing to the disease annually [1–3]. Although more than 15 TB drug candidates are currently in pre-clinical or clinical phases of drug development, new candidates are needed to supplement the drug development pipeline [3, 4]. Highly bioactive drug candidates can be selected through high-throughput screening from compound libraries, but it is difficult to select candidates for further pre-clinical trials without determining their specific modes of action (MOAs) [5–9]. It is therefore important to characterize the MOAs of bioactive molecules early in the drug discovery/development process in order to select candidates with high potential of leading to new and improved pharmaceuticals [4, 10].

**Fig. 1:**
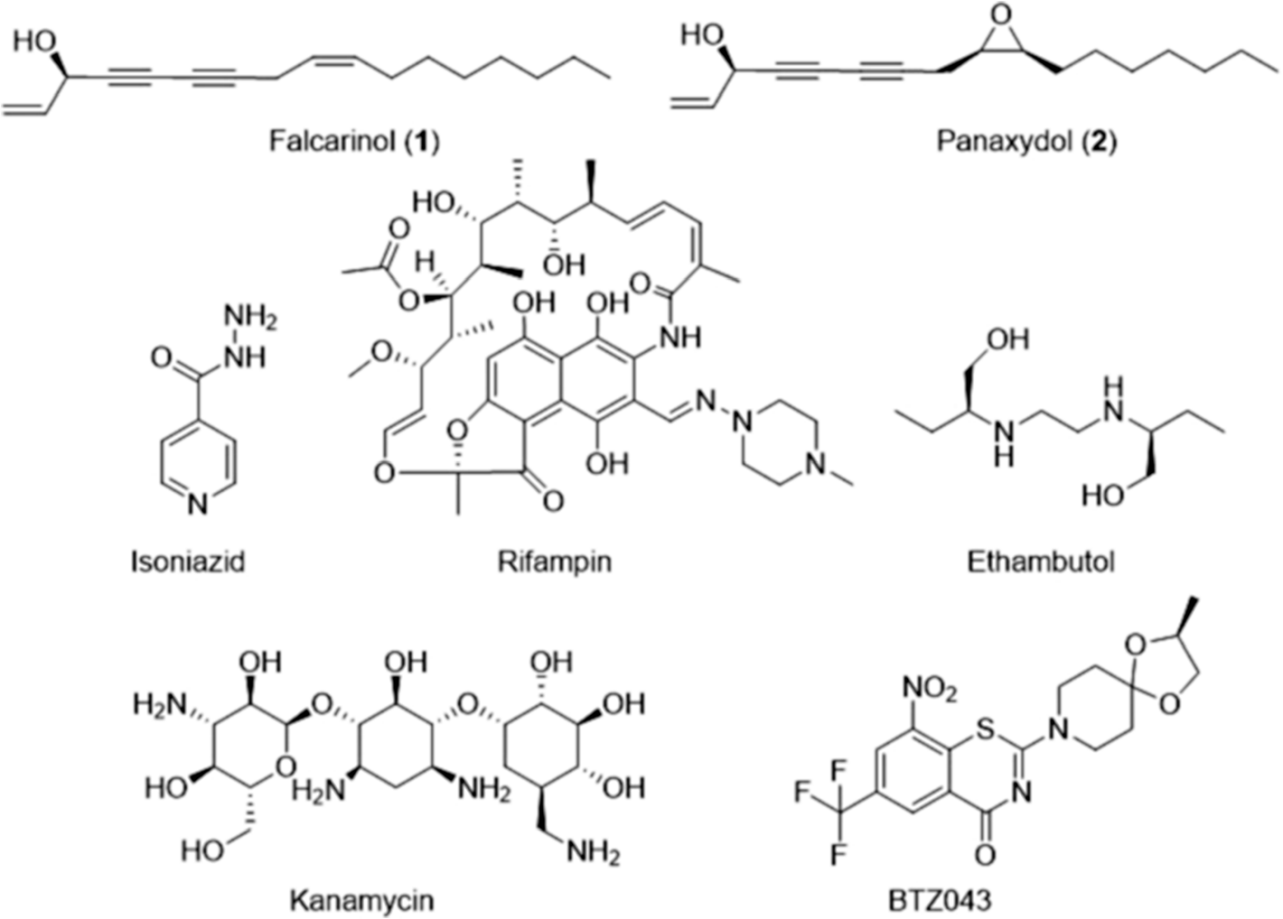
The structures of compounds mentioned in this study

Metabolic response to environmental stimuli, such as the introduction of an exogenous chemical, can trigger specific gene expression responses that are necessary for growth and survival [11–14]. Transcriptional responses can therefore provide a global view of an organism’s response to exogenous chemical stimuli [15–17]. Gene expression analysis has been used successfully to characterize the molecular targets of pharmaceuticals [14, 18, 19]. For example, microarray analysis of *Mycobacterium tuberculosis* treated with isoniazid and rifampin (Fig. 1) not only generated data that was in good agreement with the known MOAs of these TB drugs; these studies also improved the knowledge of indirect and secondary cellular responses of *M. tuberculosis* under the effects of those drugs [11, 20, 21]. Microarray data obtained from *M. tuberculosis* treated with BTZ043 (Fig. 1), a TB drug candidate in pre-clinical development, led to the discovery that BTZ043 inhibits a key enzyme in the synthesis of cell wall arabinans [22]. Thus, transcriptomic profiling can offer a great deal of insight for drug-organism interactions.

The objective of this study was to determine the transcriptome profile of *M. smegmatis* following treatment with falcarinol (**1**) and panaxydol (**2**) (Fig. 1) in order to identify the anti-mycobacterial MOAs of these compounds. Falcarinol and panaxydol were identified as anti-mycobacterial natural products from studies of the plant *Aralia nudicaulis* [23]; however, the anti-mycobacterial MOA of these two compounds was not known. Cognisant of the challenges associated with studying *M. tuberculosis* in the laboratory, we studied the closely related, non-pathogenic mycobacteria *M. smegmatis* MC^2^ 155 as a surrogate model organism [24–27]. In addition to fewer safety precautions, other benefits related to the use of *M. smegmatis* include its superior growth rate, its comparable ability to *M. tuberculosis* to produce mycolic acid, and the fact that its genome has been completely sequenced and well annotated, which facilitates transcriptomics analyses [28].

## Results and discussion

In order to explore the transcriptomic profile of *M. smegmatis* MC^2^ 155 treated with falcarinol and panaxydol, the minimum inhibitory concentrations (MICs) of the two target compounds and three positive controls, isoniazid, ethambutol and kanamycin, were measured using a modified microplate resazurin assay. The MICs of falcarinol and panaxydol against *M. smegmatis* MC^2^ 155 were 12.5 and 25 μg/mL respectively (Table 1), which is higher than their MICs against *M. tuberculosis* H37Ra (6.25 and 9.38 μg/mL respectively) [23]. Two of the positive controls, isoniazid and ethambutol, were selected because information regarding their MOA against *Mycobacterium* species is available [29, 30]. Additionally, *M. smegmatis* is naturally resistant to isoniazid because it has different peroxide stress response systems to *M. tuberculosis* [31] and the multidrug efflux pump LfrA [32], although the effect of these resistance mechanisms has not been investigated at the level of the transcriptome. The third positive control, kanamycin, was selected due to its potent activity against *M. smegmatis* [32]. The MICs against *M. smegmatis* that we obtained for of all three controls were similar to those previously reported [33, 34].

**Table 1:** Anti-mycobacterial activities of falcarinol, panaxydol, isoniazid, ethambutol and kanamycin against *M. smegmatis* MC^2^ 155

Compound	MIC (μg/mL)
Falcarinol	12.5
Panaxydol	25
Isoniazid	50
Ethambutol	1.56
Kanamycin	1.56

Microarray analysis revealed that 704 and 907 genes, or 10 and 13 % of the *M. smegmatis* transcriptome, were differentially expressed following treatments with falcarinol and panaxydol compared to the control. Using a fold change threshold of ± 2 and an α value threshold of *p* < 0.05, falcarinol and panaxydol treatments resulted in 585 and 788 differentially expressed genes (DEGs) respectively compared to the vehicle control. When comparing the two treatments, 99 % of the genes differentially expressed were in common in both treatments, evidence that the two target compounds act via the same MOA against *M. smegmatis*. All gene expression profiles from the microarray analysis are provided as supplementary data (Additional file 1).

**Fig. 2:**
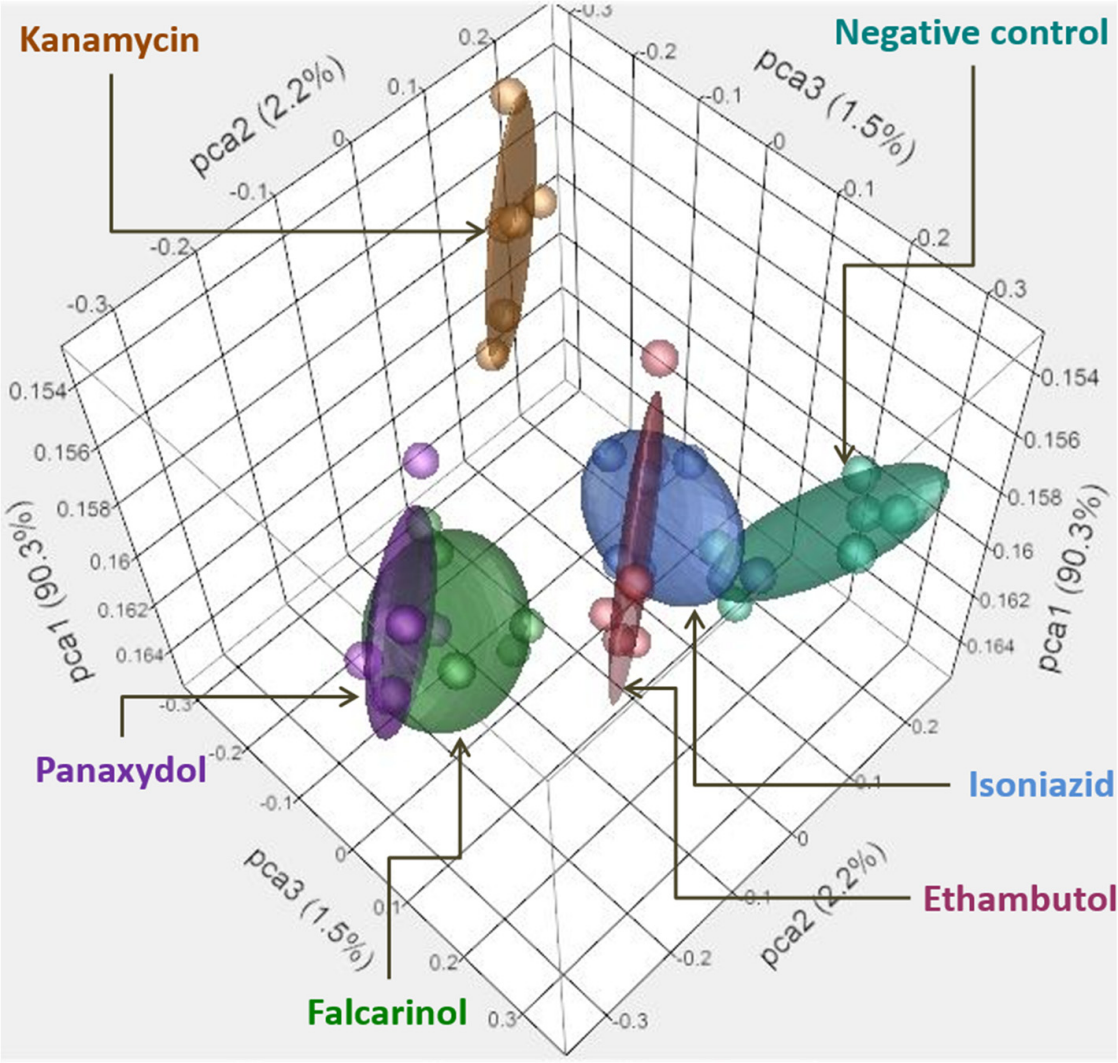
Principle component analysis of gene expression data. Each point represents one microarray conducted with one biological replicate

Principle component analysis (PCA) of all treatments revealed that exposure of *M. smegmatis* to **1** and **2** resulted in transcriptomic responses that were more similar to each other than the controls. Transcript responses to isoniazid and ethambutol treatments coincided with the vehicle control while the response to the kanamycin treatment was the most distinct of all other treatments (Fig. 2). PCA confirmed that falcarinol and panaxydol treatments resulted in relatively similar gene responses suggesting they have a common MOA. Additionally, the relative distance between the gene responses to **1** and **2** and the three positive controls in PCA provided further evidence that **1** and **2** have a MOA distinct to those of the controls; isoniazid and ethambutol both affect cell wall synthesis and kanamycin primarily targets protein synthesis (Fig. 2) [35].

The PCA provided a global view of the differences in the transcriptomic profiles of treatments; however, it does not identify what caused these differences. Therefore, we performed functional enrichment analysis to examine what biological functions may have contributed to the differences in transcriptome profiles. All of the genes in the *M. smegmatis* MC^2^ 155 genome were grouped based on their functions by assigning them to one or multiple gene ontology (GO) terms, which were grouped into three categories (molecular function, cellular component and biological processes). In order to identify the biological functions that were affected by each treatment, functional enrichment analysis was used to assess which GO terms in each treatment were significantly different to the vehicle control. The GO terms that were preferentially affected by falcarinol and panaxydol treatments were determined from P-values generated from parametric analysis of gene set enrichment (PAGE) [36] following a post-hoc analysis with a false discovery rate (FDR) of 0.01 (Table 2). Nine common GO terms were found to be significantly different to the vehicle controls in both treatments, and the processes that these represent were related to fatty acid biosynthesis, ectoine biosynthesis, and protein metabolism, transport and biosynthesis. Thus, the C17 diynes appear to affect multiple pathways within the cell and likely have different MOAs, however for our discussion we focus on the prevalent themes based on multiple lines of evidence from bioinformatics. All functional enrichment analysis data are included as supplementary data.

**Table 2:** Functional enrichment analysis of gene ontology (GO) terms regulated by falcarinol and panaxydol (P-value < 0.01 after post-hoc analysis)

Treatment	Gene ontology category	GO term	Name	Frequency	PAGE Z-Score	P-value
Falcarinol	Molecular function	go:0008658	Penicillin binding	14	5.4	3.0E-05
	Molecular function	go:0004674	Protein serine/threonine kinase activity	21	5.3	3.6E-05
	Molecular function	go:0003735	Structural constituent of ribosome	58	5.2	3.9E-05
	Biological process	go:0045227	Capsule polysaccharide biosynthetic process	7	5.0	6.3E-05
	Biological process	go:0009088	Threonine biosynthetic process	5	4.9	6.9E-05
	Biological process	go:0019491	Ectoine biosynthetic process	3	4.9	6.9E-05
	Biological process	go:0043952	Protein transport by the Sec complex	3	4.9	6.9E-05
	Biological process	go:0051188	Cofactor biosynthetic process	3	4.9	6.9E-05
	Biological process	go:0065002	Intracellular protein transmembrane transport	3	4.9	6.9E-05
	Biological process	go:0006412	Translation	67	4.4	5.0E-04
	Cellular component	go:0009360	DNA polymerase III complex	6	4.3	8.6E-04
	Cellular component	go:0005840	Ribosome	46	4.1	1.7E-03
	Molecular function	go:0003697	Single-stranded DNA binding	7	3.9	5.1E-03
	Molecular function	go:0019843	rRNA binding	36	3.7	9.5E-03
	Molecular function	go:0008825	Cyclopropane-fatty-acyl-phospholipid synthase activity	7	2.7	9.6E-03
Panaxydol	Molecular function	go:0019843	rRNA binding	36	5.7	8.1E-06
	Molecular function	go:0003735	Structural constituent of ribosome	58	5.5	8.3E-06
	Biological process	go:0006412	Translation	67	4.7	4.6E-04
	Biological process	go:0008610	Lipid biosynthetic process	9	4.5	9.3E-04
	Cellular component	go:0005840	Ribosome	46	4.4	1.1E-03
	Molecular function	go:0008825	Cyclopropane-fatty-acyl-phospholipid synthase activity	7	4.3	1.3E-03
	Biological process	go:0019491	Ectoine biosynthetic process	3	4.3	1.3E-03
	Biological process	go:0043952	Protein transport by the Sec complex	3	4.3	1.3E-03
	Biological process	go:0065002	Intracellular protein transmembrane transport	3	4.3	1.3E-03
	Molecular function	go:0004674	Protein serine/threonine kinase activity	21	3.8	9.6E-03

To further probe the pathways that were affected by **1** and **2**, a pathway enrichment analysis was performed using the Biocyc online program (http://biocyc.org/) which uses a Fisher exact test with a post-hoc application of the Benjamini-Hochberg method [37]. Differentially affected pathways identified in the falcarinol treatment included amino acid biosynthesis and fatty acid biosynthesis, such as “methionine biosynthesis” and “cyclopropane fatty acids biosynthesis”, whereas fatty acid biosynthesis was the most prevalent differentially affected pathway identified in the panaxydol treatment, such as “pyruvate fermentation to acetate” and “unusual fatty acid biosynthesis” (Table 3).

**Table 3:** Pathway enrichment analysis for pathways regulated by falcarinol and panaxydol (P-value < 0.01) after post-hoc analysis and significantly different genes related to the pathways

Treatment	Pathways	P-value	Genes in Pathway
Falcarinol	Threonine biosynthesis	5.3E-05	*MSMEG 6286, thrC, thrB, MSMEG 4957, asd, MSMEG6257*
	Ectoine biosynthesis	6.1E-05	*asd, MSMEG 6257, ectC, ectA, ectB*
	Superpathway of methionine biosynthesis (by sulfhydrylation)	4.4E-04	*MSMEG 4528, metE, metX, MSMEG 1652, MSMEG 4657, asd, MSMEG 6257, MSMEG 6286*
	Isoleucine biosynthesis I	4.4E-04	*ilvA, ilvH, thrC, thrB, MSMEG 4657, asd, MSMEG 6257, MSMEG 6286*
	Methionine Biosynthesis	2.0E-03	*metE, MSMEG 4957, asd, MSMEG 6257, MSMEG 6286, MSMEG 4528, metX, MSMEG 1652*
	Mycolyl-arabinogalactan-peptidoglycan complex biosynthesis	2.1E-03	*MSMEG 6386, MSMEG 6399, MSMEG 6382, MSMEG 6401, glf, MSMEG 4947*
	Homoserine biosynthesis	3.0E-03	*MSMEG 4957, asd, MSMEG 6257*
	Superpathway of methionine biosynthesis (transsulfuration)	4.7E-03	*metE, MSMEG 4957, asd, MSMEG 6257, MSMEG 6286*
	Unusual Fatty Acid Biosynthesis	5.5E-03	*MSMEG 1350, MSMEG 1351, MSMEG 3538*
	Cyclopropane Fatty Acids Biosynthesis	9.3E-03	*MSMEG 1350, MSMEG 1351, MSMEG 3538*
Panaxydol	Ectoine biosynthesis	3.8E-03	*MSMEG 6257, ectC, ectA, ectB*
	Urea degradation II	3.8E-03	*MSMEG 1093, ureC, ureB, ureA*
	Pyruvate fermentation to acetate VII	5.1E-03	*pta, ackA, MSMEG 4646*
	Pyruvate fermentation to acetate I	5.1E-03	*pta*
	Methionine Biosynthesis	6.3E-03	*metE, metN, MSMEG 6257, MSMEG 6286, MSMEG 4528, MSMEG 4527, metXm MSMEG 1652*
	Superpathway of methionine biosynthesis (by sulfhydrylation)	7.7E-03	*MSMEG 4528, MSMEG 4527, metE, metX, MSMEG 1652, MSMEG 6257, MSMEG 6286*
	Unusual Fatty Acid Biosynthesis	9.7E-03	*MSMEG 1205, MSMEG 1350, MSMEG 1351, MSMEG 3538*
	Cyclopropane Fatty Acids Biosynthesis	9.7E-03	*MSMEG 1205, MSMEG 1350, MSMEG 1351, MSMEG 3538*
	Urate biosynthesis/inosine 5′-phosphate degradation	9.7E-03	*MSMEG 1701, MSMEG 4308, guaB, MSMEG 1603*

Taken together, the results of the functional enrichment and pathway enrichment analysis suggest that genes coding for ectoine biosynthesis and cyclopropane-fatty-acyl-phospholipid synthesis are preferentially regulated by the treatment of **1** and **2**. Ectoine and its derivatives are compounds commonly found in bacteria that balance extracellular osmotic pressure, without altering the ionic strength of the cytoplasm [38, 39]. It has previously been demonstrated that elevated extracellular salt concentrations increase ectoine production and cause up-regulation of the ectoine biosynthetic genes [40]. Interestingly, falcarinol and panaxydol treatment of *M. smegmatis* resulted in the down-regulation of the ectoine biosynthetic genes *ectA*, *ectB* and *ectC*, suggesting that the C17 diynes do not induce osmotic stress.

Conversely, cyclopropane-fatty-acyl-phospholipid synthesis is the process by which cyclopropane rings are introduced into unsaturated mycolic acids [41, 42]. Indeed, because this process plays a crucial role in *M. tuberculosis* pathogenesis [43], cyclopropane-fatty-acyl-phospholipid synthase was used as a target for screening potential anti-TB compounds [44]. Since the two target compounds are also fatty acid derivatives that share some structural similarities with precursors of cyclopropane phospholipid synthesis, we postulate that **1** and **2** act as competitive inhibitors of cyclopropane-fatty-acyl-phospholipid synthase that disrupt mycolic acid metabolism. Inhibition of mycolic and fatty acid biosynthesis is an acknowledged mechanism by which compounds can exhibit anti-mycobacterial activity [45] and inhibitors of these pathways have been shown to exert their effects on both *M. tuberculosis* and *M. smegmatis* [46]. Natural products that contain long aliphatic hydrocarbon chains have been demonstrated to be fatty acid synthesis inhibitors in *Mycobacterium* spp*.* in previous studies. A mixture of two fatty acids isolated from a Turkish sponge showed significant activity (IC_50_ = 0.35 μg/mL) against the enzyme *Fab I*, which is essential for type II fatty acid biosynthesis in *M. tuberculosis* [47]. An acetylenic thiolactomycin isolated from a soil bacterium, *Nocardia* spp*.*, was found to inhibit the *Fab H* fatty acid condensing enzymes, *mtFab H* and *Kas A*, in *M. tuberculosis* [48]. These two examples support the hypothesis that the two C17 diynes are fatty acid synthesis inhibitors; however, additional experimental data are needed to support or refute this hypothesis.

To corroborate the results of our microarray analyses and confirm that the molecular targets of the two C17 diynes are involved in lipid biosynthesis, we used the Prediction of the Biological Activity Spectra of Organic Compounds (PASS) program to explore the prospective biological activities of **1** and **2** from their chemical structures. PASS has been used to predict successfully MOAs of anti-mycobacterial natural products from various sources [49]. All of the activities that related to lipid biosynthesis were contained in the PASS output and the majority of these were related to phospholipid biosynthesis (Table 4). The complete list of predicted biological activities of **1** and **2** are presented as supplementary information. There is therefore excellent congruence between the predicted activities of the diynes based on their structure and our transcriptomic data that identifies lipid biosynthesis as the likely pathway affected by these compounds.

**Table 4:** Predicted fatty acid synthesis related MOAs of falcarinol and panaxydol using PASS program

Treatment	Pa^a^	Pi^b^	Biological activity
Falcarinol	8.2E-01	8.0E-03	Alkyl-acetyl-glycero-phosphatase inhibitor
	8.0E-01	5.0E-03	Fatty-acyl-CoA synthase inhibitor
	6.9E-01	1.2E-02	Lipid metabolism regulator
	6.2E-01	9.0E-03	Phosphatidyl-glycero-phosphatase inhibitor
	4.5E-01	4.3E-02	Alkenyl-glycero-phospho-ethanolamine hydrolase inhibitor
	3.6E-01	1.3E-02	Cyclopropane-fatty-acyl-phospholipid synthase inhibitor
Panaxydol	6.7E-01	2.4E-02	Alkyl-acetyl-glycero-phosphatase inhibitor
	6.7E-01	1.6E-02	Fatty-acyl-CoA synthase inhibitor
	6.6E-01	1.4E-02	Lipid metabolism regulator
	3.4E-01	3.6E-02	Phosphatidyl-glycero-phosphatase inhibitor
	3.8E-01	6.5E-02	Alkenyl-glycero-phospho-ethanolamine hydrolase inhibitor
	2.7E-01	3.7E-02	Cyclopropane-fatty-acyl-phospholipid synthase inhibitor

^a^ Probability to be active

^b^ Probability to be inactive

The positive controls, isoniazid, ethambutol and kanamycin, with partially or fully known MOAs, were used as comparison compounds for the C17 diynes. In order to further validate the hypothesised MOA of **1** and **2** that was generated from microarray data, it is necessary to discuss the transcriptome profiles of the positive controls in relation to their partially or fully known MOAs. Isoniazid is thought to inhibit the NADH-dependent enoyl acyl carrier protein reductase (*InhA*) in the fatty acid synthase type II (FAS-II) pathway leading to depletion of mycolic acids in cell wall synthesis [50]. The *kas* operon, a set of five FAS-II genes, showed significant up-regulation in the isoniazid treatment of *M. smegmatis* (Additional file 2: Table S1), which is consistent with previous studies of *M. tuberculosis* treated with isoniazid [20, 51, 52]. In addition, similar to isoniazid treated *M. tuberculosis* [20], the main target of isoniazid, *inhA*, did not show induction in this microarray analysis and the reason for this observation, both by us and by others, remains unclear. Similar to isoniazid, ethambutol targets mycobacterial cell wall synthesis. However, instead of targeting the fatty acid component of cell wall synthesis, ethambutol inhibits arabinosyl transferases by disrupting mycobacterial cell wall synthesis [29]. From the pathway enrichment analysis, ethambutol treatment differentially affected mycolyl-arabinogalactan-peptidoglycan complex biosynthesis, heme biosynthesis, and cell structure biosynthesis (Additional file 3), whereas a previous proteomic analysis of ethambutol treated *M. smegmatis* also showed induction of transmembrane alanine and lysine rich protein in peptidoglycan biosynthesis and several proteins in the heme biosynthesis pathway [27]. In contrast to isoniazid and ethambutol, kanamycin targets the 30S subunit of prokaryotic ribosomes [53]. Interestingly, many of the genes coding for 30S or 50S ribosomal proteins showed significant down-regulation in this study (Additional file 2: Table S2). This observation may be explained by the high dosage of the treatment, which may have led to the complete inhibition of protein translation processes. Moreover, the PAGE analysis showed that GO terms, such as protein targeting, regulation of cell shape, protein transport by the sec complex, cell wall macromolecule biosynthetic processes and intracellular protein transmembrane transport (Additional file 4), were significantly affected by kanamycin treatment. It provided additional evidence for the cause of cell death by kanamycin, which is triggered by mis-translation of the membrane protein [54]. Therefore, there was congruence between the microarray data and the MOAs of these well-characterized antibiotics, increasing confidence that the microarray analyses of **1** and **2** provide insight into their MOA.

**Fig. 3:**
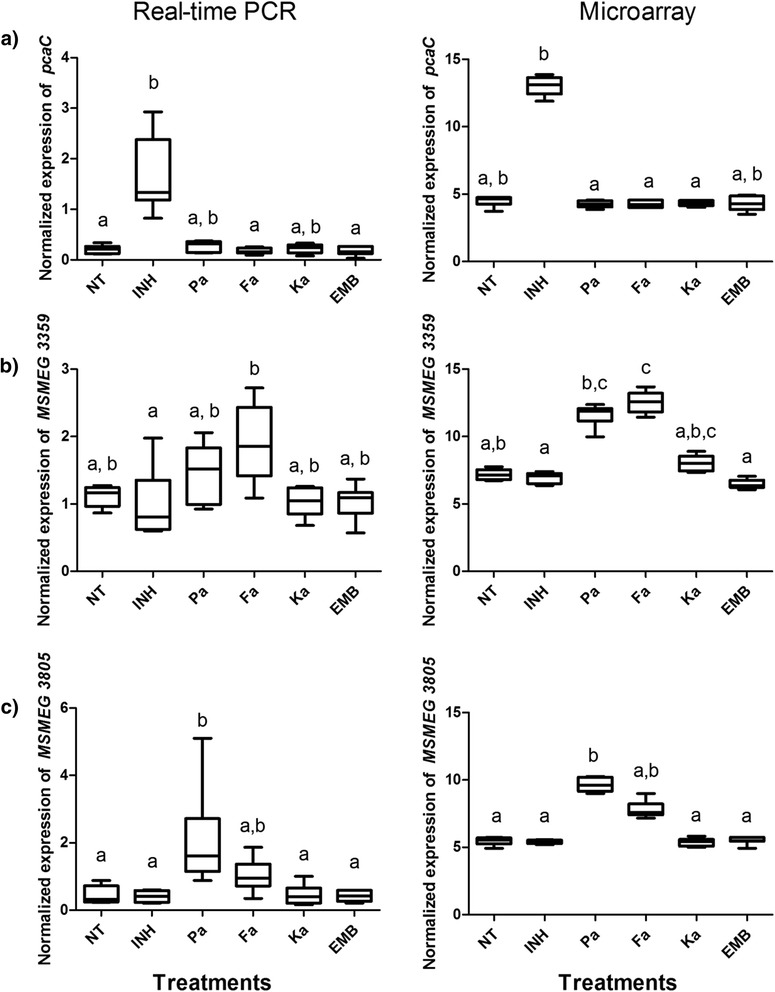
Real time PCR comparison to expression data from the microarray (log2) for (A) *pcaC* (B) *MSMEG 3359* and (C) *MSMEG 3805*. The horizontal line in the box plots represents the median of the group, the boundaries of the box represent the 10^th^ and 90^th^ percentiles and the minimum and maximum data points are represented by the whiskers (Prism v5.0). Differences among groups were tested using a Kruskal Wallis test with Dunn method for joint ranking. The small case letters “a”, “b” and “c” were used to represent different groups. NT = negative treatment, INH = isoniazid, Pa = panaxydol, Fa = falcarinol, Ka = kanamycin, EMB = ethambutol

Microarray data were further verified by real-time polymerase chain reaction (PCR). Expression data were analyzed using the Kruskal-Wallis test with Dunn’s method for joint ranking. *MSMEG 3359, MSMEG 3805* and 4-carboxymuconolactone decarboxylase (*pcaC*) showed expression patterns across each group that were consistent with the microarray data (Fig. 3). The real-time PCR and microarray data were significantly correlated in a linear regression model (Additional file 2: Figure S1). The gene *pcaC* is involved in the catabolism of protocatechuate to succinate and acetyl-CoA in the beta-ketoadipate pathway [55]. The increased expression of *pcaC* by the treatment of INH, which confers natural resistance of *M. smegmatis* against INH, may be a compensatory response to increase production of acetyl-CoA [56]. The mRNA levels of both *MSMEG 3359 and MSMEG 3805* were higher in the panaxydol treatments compared to the negative control and isoniazid, suggesting that these genes are involved in the response to these compounds, however, functional data for *MSMEG 3359 and MSMEG 3805* are lacking in the literature. The amplicons of the genes used in real-time PCR are provided in the supplementary data (Additional file 2: Table S3).

## Conclusion

Using microarray analysis and PASS, we hypothesize that both falcarinol and panaxydol inhibit phospholipid biosynthesis in mycobacteria and suggest that further studies should be directed at supporting or refuting this hypothesized MOA. To test this hypothesis, the enzyme(s) that **1** and **2** act upon should be isolated and an enzyme inhibition assay performed to measure the binding affinity of falcarinol and panaxydol to the enzyme. Cognisant that there are likely additional mechanisms of action for the C17 diynes within the cell that may not be reflected in a transcriptomic response, further studies should also include additional methods to elucidate the full effect of these compounds.

## Methods

### *Mycobacterium smegmatis* growth condition and treatments

*Mycobacterium smegmatis* strain MC^2^ 155 (American Type Culture Collection [ATCC] 700084) was grown in Middlebrook 7H9 (Becton Dickinson, Mississauga, Ontario [ON], Canada) broth supplemented with 10 % albumin dextrose catalase (ADC) enrichment (Becton Dickinson, Mississauga, ON, Canada) and 0.2 % glycerol at 37 °C for 72 h in a humid environment before being diluted to a turbidity equivalent to a 1.0 McFarland standard (10^7^ colony forming units [CFU]) using the same modified 7H9 broth and cryogenically preserved (−80 °C; 1.5 mL aliquots). Cryopreserved *M. smegmatis* was thawed and diluted with modified Middlebrook 7H9 broth (1:5) resulting in the mycobacterial suspension (2 × 10^6^ CFU) used for the bioassays.

The MIC of target treatments (**1** and **2**) and positive controls (isoniazid, ethambutol and kanamycin) were measured using a *M. smegmatis* bioassay, which was modified from a microplate resazurin assay previously developed in our laboratory [57] by changing the resazurin addition time and total incubation time from 72 and 96 h to 24 and 48 h, respectively.

To prepare cultures of *M. smegmatis* for gene expression analysis, cyropreserved *M. smegmatis* suspensions were diluted as described above and transferred to 96 well plates. The plates were first incubated for 48 h to reach log phase growth and then each treatment, including one vehicle control, three positive controls and two target compounds, was added to corresponding rows of a 96 well plate except the peripheral wells resulting in 10 wells per treatment. The vehicle control consisted of modified 7H9 broth with 2 % dimethyl sulfoxide (DMSO), whereas the concentrations of positive controls and target compounds were 10 × MIC for each treatment against *M. smegmatis*. The plates were incubated at 37 °C for 6 h in a humid environment. The bioassay time point and doses were chosen based on previously published studies [11, 20, 21]. Following 6 h incubation, wells containing the same treatment in each plate were pooled into a 1.5 mL centrifuge tube and bacteria were harvested by centrifugation. The vehicle control and two positive controls (isoniazid and ethambutol) each had six biological replicates; the two target treatments (falcarinol and panaxydol) and one additional positive control (kanamycin) each had seven biological replicates. All biological replicates were used for microarray analysis and real-time PCR.

### RNA extraction

Total ribonucleic acid (RNA) was extracted using TRIzol® (Invitrogen, Burlington, ON, Canada) and purified using an RNeasy® kit (Qiagen, Toronto, ON, Canada) according to the manufacture’s protocol. RNA quantity was measured on a NanoDrop ND 2000 spectrometer (Thermo Scientific, Wilmington, DE, USA) before and after purification. RNA quality was evaluated using an Agilent 2100 Bioanalyzer (Agilent, Mississauga, ON, Canada). All samples used in the microarray and real-time PCR experiments had RNA integrity numbers (RINs) > 7.5.

### Microarray analysis and bioinformatics

*Mycobacterium smegmatis* 8 × 15 K Agilent microarrays (designed by Genotypic Technology, Bengaluru, India and manufactured by Agilent, Santa Clara, CA, USA) was used to investigate global mRNA profiles of *M. smegmatis* treated with **1** and **2**. RNA labeling, microarray hybridization, and microarray scanning were conducted as directed in the Agilent one-colour microarray-based gene expression analysis protocol. Raw expression data along with *tif* images were extracted using Agilent Feature Extraction Software (v10.7.3.1). All microarray data reported in this study follow established guidelines (i.e. Minimum Information about a Microarray Experiment http://www.ncbi.nlm.nih.gov/geo/info/MIAME.html) and have been deposited in the National Center for Biotechnology Information (NCBI) Gene Expression Omnibus (GEO) database (GSE64323, GPL19567).

The raw expression data were normalized using Loess normalization (smoothing factor of 0.2). The limit of detection of the microarray normalized signals was determined to be 3.5 based on the lower limit of the standard curve for Agilent quality controls and negative controls (dark corners) so all intensity values that were lower than 3.5 were assigned a value of 3.5. DEGs were determined using one-way analysis of variance (ANOVA) followed by a post-hoc test using Benjamini and Hochberg method with a FDR at 0.01. PCA was performed on all transcripts using normalized expression values. Functional enrichment of GO terms was performed using PAGE analysis [36] followed by a post-hoc test using Benjamini and Hochberg method with FDR set at 0.01. The above data processing and bioinformatics analysis were performed in JMP® Genomics (v5.1).

DEGs (adjusted P-value < 0.01) obtained from ANOVA was uploaded to Biocyc online program (http://biocyc.org/) [58], and pathway enrichment analysis was performed using Fisher’s extract test with Benjamini-Hochberg [59] method as a post-hoc analysis.

### cDNA synthesis, primer design and real-time PCR

The complementary deoxyribose nucleic acid (cDNA) synthesis, primer design and real-time PCR were performed as previously reported [60]. Primers used in this study are listed in Table 5. Mean expression levels of *MSMEG 3584*, *MSMEG 5570* and *gidB* were determined to be the most stable combination of control genes to normalize all target expression data, with a mean M-value of 0.67 (mean coefficient variance = 0.25) as determined by the target stability function in CFX96 software. Normalized gene expression values were extracted using a relative ∆∆Cq method in CFX Manager™ 3.0 software (BioRad, Mississauga, ON, Canada). Normalized expression data were not normally distributed and a Kruskal–Wallis non-parametric test was used to determine if gene expression levels varied across treatments and a non-parametric Dunn method for joint ranking was used as a post-hoc test to determine which treatments were different from each other. All statistical analyses were conducted in GraphPad Prism 5.0.

**Table 5:** Primer sequences used for real-time PCR with amplicon size and annealing temperature. All primer sets had R^2^ > 0.98 and efficiency percentage between 90 % and 110 %

Gene	Forward primer (5′-3′)	Reverse primer (5′-3′)	Product size (bp)	Annealing (°C)
*alkB (MEMEG 1839)*	GCCTACATCCCGTTCCAGT	AGCGAGTCCTTCTTGTGTCC	191	58
*ectB (MSMEG 3900)*	AGGATTTTTCCGTGTTGCTC	TTGTTGTGCCCGTAGTTCAG	214	58
*katG (MSMEG 3461)*	GCCACCCAGGAAGAGACC	GCAGGTTGACGAAGAAGTCC	265	58
*mmpl5 (MSMEG 1382)*	CGAATCTGGCTACCTGTGCT	GTGGCGGTCCTCTCTTCTTT	365	58
*MSMEG 3359*	CACCGACATACACTGCCAAC	GAACCACGCCTTCTCCTG	310	58
*MSMEG 3805*	GGGGAGCCATTCTCAACG	TGTGTTCCTCGGGCAGTTC	225	59
*pcaC (MSMEG 6370)*	CGAGACCGAGCAGCGACT	CGGGAACGGCATCTTCAC	250	59
*gidB (MSMEG 6940)* ^1^	ATGCCAGGGTGGTGAGAT	CGTGAAACATTCGGCTTCT	270	61
*MSMEG 3496*	TATGAGCGTGGTGGTCCTG	GCGGTCGTTGTAGTTGGTCT	229	61
*MSMEG 3584* ^a^	TGTCGGAGTTGTTGATGGTC	CTGTCGGTGTTCTCGTTCAG	241	61
*MSMEG 5570* ^a^	CACCGAGAAAGAACTGAGCA	GCAACTATTCCCACACAACCT	173	60

^a^ Genes used as normalizer gene

### Prediction of biological activities

The structures of **1** and **2** were converted to simplified molecular-input line-entry system (SMILES) format and uploaded to Prediction of the biological activity spectra of organic compounds (PASS) online program (http://www.way2drug.com/PASSOnline) for biological activity prediction [61]. A master list, which entails all potential bioactivities of the two target compounds and probabilities of each bioactivity being active or inactive, was generated through the PASS online program (Additional file 5) [61].

## Abbreviations

ADC, albumin, dextrose, catalase; ANOVA, analysis of variance; ATCC, American Type Culture Collection; cDNA, complementary deoxyribose nucleic acid; CFU, colony forming units; DEG, differentially expressed gene; DMSO, dimethyl sulfoxide; FDR, false discovery rate; GEO, Gene Expression Omnibus; GO, gene ontology; MIC, minimum inhibitory concentration; MOA, mode of action; NCBI, National Center for Biotechnology Information; ON, Ontario; PAGE, parametric analysis of gene set enrichment; PASS, prediction of the biological activity spectra of organic compounds; PCA, principle component analysis; PCR, polymerase chain reaction; RIN, RNA integrity number; RNA, ribonucleic acid; SMILES, simplified molecular-input line-entry system; TB, tuberculosis

## Additional files

Additional file 1: ANOVA results for all transcripts in each treatment group compared to vehicle control. (XLSX 24787 kb) Additional file 2: Supplementary data; Figure S1. Correlation of fold change (log10) between real-time PCR and microarray data; Table S1. Sequenced genes and their amplicons in *M. smegmatis*; Table S2. Response of the *kas* operon in *M. smegmatis* to isoniazid treatment compared with vehicle control; Table S3. Fold changes of significantly different ribosomal protein genes from *M. smegmatis* treated with kanamycin. (DOCX 91 kb) Additional file 3: Enriched pathways for each treatment group. (XLSX 32 kb) Additional file 4: Enriched GO terms for each treatment group. (XLSX 231 kb) Additional file 5: Predicted bioactivities for falcarinol and panaxydol from PASS. (XLSX 81 kb)
