# Supplementary material for: Determining the mode of action of anti-mycobacterial C17 diyne natural products using expression profiling: evidence for fatty acid biosynthesis inhibition
Source: BMC Genomics. 2016 Aug 11;17:621. doi: 10.1186/s12864-016-2949-y (PMC4981992; doi:10.1186/s12864-016-2949-y)
Supplement: Additional file 2: — Supplementary data; Figure S1. Correlation of fold change (log10) between real-time PCR and microarray data; Table S1. Sequenced genes and their amplicons in M. smegmatis; Table S2. Response of the kas operon in M. smegmatis to isoniazid treatment compared with vehicle control; Table S3. Fold changes of significantly different ribosomal protein genes from M. smegmatis treated with kanamycin. (DOCX 91 kb) [file 12864_2016_2949_MOESM2_ESM.docx]

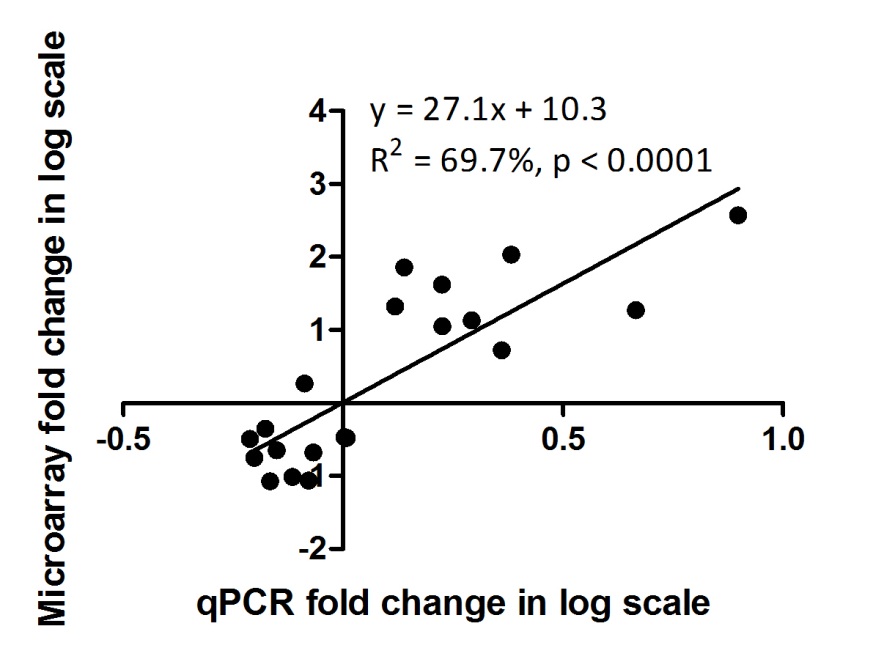


**Figure S1 Correlation of fold change (log10) between real-time PCR and microarray data.** The best fit was calculated by linear regression. Each dot represents the coordinates of the fold change for qPCR and microarray for the same gene. The fold changes are the ratios of normalized expression of treatments and vehicle control in qPCR and microarray data. A data point was used only if it showed a significant change in real-time PCR, microarray, or both (P<0.05).

**Table S1 Response of the *kas* operon in *M. smegmatis* to isoniazid treatment compared with vehicle control.** Fold change was calculated from normalized microarray expression. P-value is the FDR post-hoc correction after ANOVA.

| *kas* operon | *fabD* | *acpM* | *kasA* | *kasB* | *accD6* |
| --- | --- | --- | --- | --- | --- |
| Fold changes | 2.6 | 2.4 | 2.4 | 2.3 | 2.2 |
| P-values | 1.8E-06 | 3.7E-06 | 1.8E-06 | 6.0E-05 | 7.5E-05 |

**Table S2 Fold changes of significantly different ribosomal protein genes from *M. smegmatis* treated with kanamycin**. Fold change was calculated from normalized microarray expression. P-value is the FDR post-hoc correction after ANOVA.

| Gene | transcripts | Fold change | P-value |
| --- | --- | --- | --- |
| *rpsC* | 30S ribosomal protein S3 | -3.5 | 7.2E-07 |
| *rplP* | 50S ribosomal protein L16 | -5.0 | 1.7E-06 |
| *rplQ* | 50S ribosomal protein L17 | -2.5 | 3.4E-06 |
| *rpmC* | 50S ribosomal protein L29 | -4.0 | 2.0E-05 |
| *rplS* | 50S ribosomal protein L19 | 1.9 | 2.2E-05 |
| *rpsS* | 30S ribosomal protein S19 | -2.5 | 3.1E-05 |
| *rplU* | 50S ribosomal protein L21 | 1.9 | 3.3E-05 |
| *rpmA* | 50S ribosomal protein L27 | 1.8 | 3.6E-04 |
| *rpsQ* | 30S ribosomal protein S17 | -3.5 | 1.6E-03 |

**Table S3 Sequenced genes and their amplicons in *M. smegmatis*.** Nucleotide sequences from all amplicons were aligned with the genes that were used to design the primers by pairwise sequence alignment tool (EMBOSS needle) and all nucleotides have a 95% or greater match to the amplification region of the corresponding gene.

| Gene | Sequencing |
| --- | --- |
| *alkB (MEMEG 1839)* | cggcggtctgggctggcccgccaagatcggcctggcgctgtcggtcggcgtgctcggcggggtcggcatcaacaccgcccacgaactcggacacaagaagg |
| *ectB (MSMEG 3900)* | gcaccgtcgagtccgaggtccgtagctactgccgcgggtggcccgcggtcatggagaccgcgaaggactcgtgggtgaccgacgtcgacggtcgccgctacatcgacttcttcgcgggcgccggcgcgctgaactacggg |
| *katG (MSMEG 3461)* | cctgggcaagggttcggacctgcccgcggagttcaagctgatcgaccgggcgaacctgctgggcctgtcggctcctgagatgaccacgctcgtcggcggtctgcgggtgctcgacgtcaaccacggcggtaccaagcacggcgtgctgaccgacaagccgggcgcgttgaccacggacttctt |
| *mmpl5 (MSMEG 1382)* | cctgggcaagggttcggacctgcccgcggagttcaagctgatcgaccgggcgaacctgctgggcctgtcggctcctgagatgaccacgctcgtcggcggtctgcgggtgctcgacgtcaaccacggcggtaccaagcacggcgtgctgaccgacaagccgggcgcgttgaccacggacttctt |
| *MSMEG 3359* | cgagggcgattgcttcgtcgcacgcaagcctgaagggcgttcctttctgggtggccagatccggcacgggcgctccgtggaaacacgccaggcgatgctgaaagcgttgcgggacatgtgggtgcagaccacgggccaatccgaggccgagctcatcgtcggcatatccgaagtggatccccgaatggtgctagaagcgggattcttcatgccggagccgggccag |
| *MSMEG 3805* | gtcctatgccgccgcgaatccgcggaatgggggttgaccaacggtgttcgcgtggaactcgcgcgccagggcacccacgtcgccgctctggtacccgggctgatccgcaccgacacgctgctcgagttcgcccgcggcaacgggatcgaactgcNNgaggaacac |
| *pcaC (MSMEG 6370)* | aggcggctctcgcgctcaccgaggcggtgacggagatcaaccgcggcccggtgtctgacgacgtctacgaacgtgcggccgcggtgttcagcgaacgcgaactcagtcagttgatcgcgatgattgtgaccatcaacgcgtggaaccgcatcaacgtcaccgtga |
| *gidB (MSMEG 6940)* | gccgtacgNNNNcaNNcacggcggcgggcggNNNaccgggacggaacaagtcgggtcggacggcgcggtcccggggaagaacgggcaggagatgagcatgggttcgggtcagaacaaaggacagggcgtggtgcagaagccgaNNgtttcacg |
| *MSMEG 3496* | gacacgcgatggtggtcaccaggatcggcgcaggccaacgcaccacggccgcaccgagtttgcgccacccgcgggNNNgcatcgcacgcttgggctcgaaggtcttgccgaaccggctcgcgaccgagatgatcgcggcgcccatggtcaacgaggccaggaccacNaNNNNca |
| *MSMEG 3584* | gNNNNNtctcccttgaccgggcggcgaaatccctcatccggcttcccggtgtggccatggtgcagagcatcaccagaccactgggccggccactggagcacgcgaccattccctacctgttcaccatgcagggcagcaccagcggccagcaactgccgttcgaccaacaactgaacgagaacaccgaca |
| *MSMEG 5570* | ttttcNacNNNNNctcagcatcgcgcaggtgatggtgccgcacatcgccgggcggccggtgacgcgcaaacgctggcccaacggcgtcgcggaagaggcgttcttcgagaagcagttggcgt |
